# Impaired Self-Awareness in Parkinson’s and Huntington’s Diseases: A Literature Review of Neuroimaging Correlates

**DOI:** 10.3390/brainsci14030204

**Published:** 2024-02-23

**Authors:** Manuela Tondelli, Miriana Manigrasso, Giovanna Zamboni

**Affiliations:** Department of Biomedical, Metabolic and Neural Science, University of Modena and Reggio Emilia, 41121 Modena, Italy

**Keywords:** self-awareness, Parkinson’s Disease, Huntington’s Disease, MRI, PET neuroimaging

## Abstract

Little is known about the brain correlates of anosognosia or unawareness of disease in Parkinson’s Disease (PD) and Huntington’s Disease (HD). The presence of unawareness or impaired self-awareness (ISA) of illness has profound implications for patients and their caregivers; therefore, studying awareness and its brain correlates should be considered a key step towards developing effective recognition and management of this symptom as it offers a window into the mechanism of self-awareness and consciousness as critical components of the human cognition. We reviewed research studies adopting MRI or other in vivo neuroimaging technique to assess brain structural and/or functional correlates of unawareness in PD and HD across different cognitive and motor domains. Studies adopting task or resting-state functional magnetic resonance imaging, and/or 18-F fluorodeoxyglucose positron emission tomography brain imaging and/or magnetic resonance imaging structural measures were considered. Only six studies investigating neuroimaging features of unawareness in PD and two in HD were identified; there was great heterogeneity in the clinical characteristics of the study participants, domain of unawareness investigated, method of unawareness assessment, and neuroimaging technique used. Nevertheless, some data converge in identifying regions of the salience and frontoparietal networks to be associated with unawareness in PD patients. In HD, the few data are affected by the variability in the severity of motor symptoms. Further studies are needed to better understand the mechanisms and brain correlates of unawareness in PD and HD; in addition, the use of dopaminergic medications should be carefully considered.

## 1. Introduction

The first clinical description of poor awareness of illness can be traced back to 19th-century reports depicting the “belle indifference” in hysteria [1] and the “einsichtslos” described in psychosis by von Kraft-Ebing in 1878, defining with those terms the patient’s inability to recognize their delirious condition. Anosognosia (*a*, without; *noso*, disease; *gnosia*, knowledge) was a term firstly introduced in 1914 by Babinski to describe the clinical syndrome of the unawareness of hemiplegia of the left side of the body due to damage to the right cerebral hemisphere [2]. Its use has been subsequently extended to other neurological symptoms, including cognitive impairment, and defined as the condition of a patient affected by a brain dysfunction who does not recognize the presence or adequately appreciate the severity of deficits in sensory, perceptual, motor, affective, or cognitive functioning evident to clinicians and caregivers [3,4].

The presence of unawareness or impaired self-awareness (ISA) of illness has profound implications for patients and their caregivers. Patients with anosognosia may resist treatment interventions because they are not aware of their deficits; they may also experience problems in daily life functioning where they can be at risk of harming themselves or others because they cannot judge situations adequately. Furthermore, unawareness of illness has been associated with caregiver burden and delayed diagnosis by preventing patients from seeking medical evaluation [5]. From a more theoretical point of view and research perspective, the study of the neuropsychological and psychological mechanism underlying unawareness of neurological disorders offers a window into the mechanism of self-awareness and consciousness as critical components of human cognition and may contribute to a better understanding of the pathogenesis of the disorder [5,6,7,8]. Unawareness of illness in neurodegenerative diseases has mainly been studied from a neuropsychological point of view. However, if we consider unawareness as the result of an imbalance between brain damage and attempt to adapt responses to brain dysfunction, identifying the specific brain structures involved is important for understanding the underlying mechanisms of unawareness. Most studies have investigated the neuropsychological correlates of anosognosia in the Alzheimer’s Disease (AD) continuum [4,9], showing that anosognosia in Mild Cognitive Impairment (MCI) and AD reflects, at least in part, a failure to update the enduring self-awareness system based on the set of beliefs of one’s own capacities, attitudes, and traits in relation to those of others [10]. Several imaging studies of anosognosia have mainly looked at correlations between clinical measurements of anosognosia and imaging variables capturing brain metabolism (such as 18-F fluorodeoxyglucose positron emission tomography, FDG-PET), brain morphology (such as volumetric MRI) [7,8,11], and functional connectivity [12]. Critical areas including medial frontal, medial parietal and lateral parieto-temporal regions emerged consistently when reviewing studies of awareness brain correlates in AD [11,13], emphasizing the role of impaired connectivity in crucial nodes of large-scale cognitive networks in awareness processes.

On the contrary, less is known about features of anosognosia or impaired self-awareness in other neurodegenerative diseases, particularly in Parkinson’s Disease (PD) and Huntington’s Disease (HD). In patients with Parkinson’s Disease (PD), reduced or impaired self-awareness of motor symptoms (ISAm) has been reported in up to 66% of patients [14,15]. PD awareness studies mainly focused on dyskinesia, especially levodopa-induced dyskinesia (LID) [16,17,18], and on hypokinetic motor deficits [15]. Instead, awareness of cognitive and neuropsychiatric symptoms (ISAcog) has rarely been investigated in PD. Traditionally, patients with PD were thought to be aware of their cognitive difficulties, especially when compared to patients with AD; however, the recent literature demonstrated the presence of impaired self-awareness in PD for executive dysfunction and memory deficits as well for neuropsychiatric symptoms (see [19] for a review), even if these findings are mixed and limited by methodological heterogeneity. In Huntington’s Disease, unawareness is still understudied in comparison to both AD and PD. However, it is a common clinical observation that patients with HD may have limited insight into their motor, behavioral, and cognitive deficits [20]. Some authors have suggested that the limited awareness of motor impairments, especially regarding chorea, is a prominent characteristic of Huntington’s Disease in its early stages [21,22].

In summary, while individual studies may have drawn different conclusions about the brain correlates of unawareness in PD and HD, reviewing the studies within a unique context allowed for clarification of what is currently known about the brain correlates of unawareness in PD and HD. The aim of this review was to provide an overview of existing knowledge on the brain correlates of unawareness in PD and HD; we reviewed research studies that adopted MRI or other in vivo neuroimaging techniques to assess the brain’s structural and/or functional correlates of unawareness in PD and HD across different cognitive and motor domains. To the best of our knowledge, there are no published reviews of the existing literature on the neuroimaging correlates of impaired self-awareness in Parkinson’s and Huntington’s diseases; other reviews have mainly focused on clinical features of motor and cognitive/neuropsychiatric awareness, without a specific interest in their neural correlates [18,19,20,23,24]. Understanding awareness mechanisms and their associated neural correlates in PD and HD is a key step towards developing the effective management of this symptom.

## 2. Materials and Methods

### 2.1. Inclusion and Exclusion Criteria

Articles were selected according to predefined inclusion criteria: (a) studies focusing on awareness, both for motor and cognitive/neuropsychiatric symptoms; (b) studies focusing on Parkinson’s Disease and Huntington’s Disease; (c) studies using in vivo neuroimaging techniques to assess the brain’s structural and/or functional correlates of unawareness (magnetic resonance imaging, 18-F fluorodeoxyglucose positron emission tomography, functional connectivity); (d) studies of humans; (e) studies available in English and as full-text. Exclusion criteria were as follows: (a) articles that did not include neuroimaging technique as primary method of investigation; (b) articles not related to awareness for motor or cognitive/neuropsychiatric symptoms; (c) studies of animals or other populations; (d) reviews and meta-analyses; (e) non-full-article studies (including, but not limited to, conference proceedings or anecdotal reports).

### 2.2. Search Strategy

**Figure 1:**
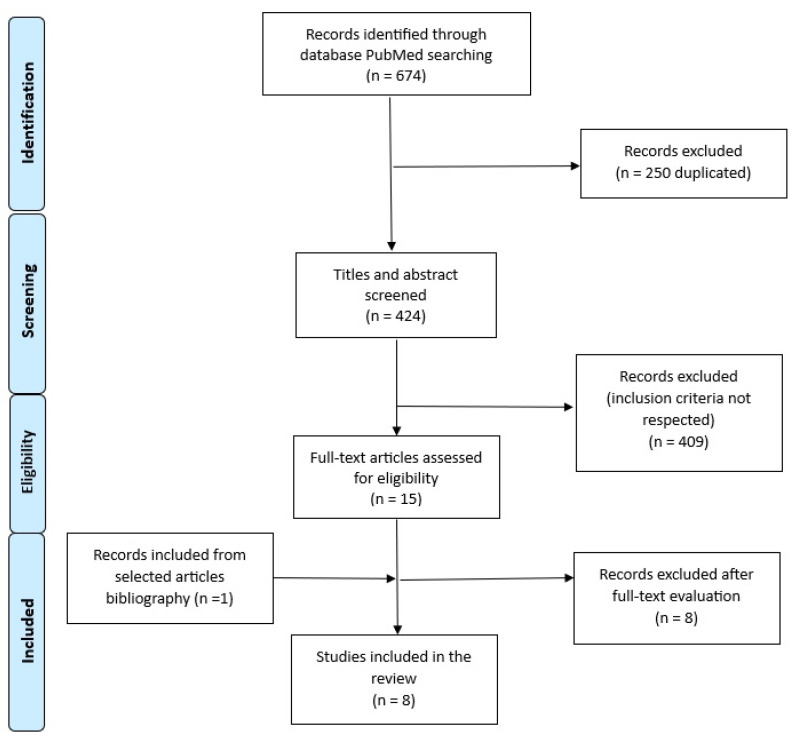
Flow-chart of study selection. The diagram shows the detailed process of study selection according to the PRISMA guidelines for systematic reviews and meta-analyses.

In order to capture the extent of the literature, a range of search terms were used. Either “Parkinson’s” or “Huntington’s” were used as a term to denote the disease of interest, combined with either “self-insight”, “anosognosia”, “unawareness”, or “self-awareness” to denote the neuropsychological area of interest and “MRI”, “resonance”, “imaging”, or “PET” to denote neuroimaging data. Literature searches were performed using the PubMed Medline, Scopus, Embase, and Web of Science databases. The PubMed search’s final line was the following: [((anosognosia) OR (unawareness) OR (self-awareness) OR (self-insight)) AND ((Parkinson) OR (Huntington)) AND ((MRI) OR (resonance) OR (imaging) OR (PET))]. The Embase search’s final line was the following: Parkinson AND (‘awareness’/exp OR awareness OR ‘anosognosia’/exp OR anosognosia OR ‘insight’/exp OR insight) AND (‘mri’ OR ‘pet’ OR ‘resonance’). We retrieved research studies published from inception to 31 December 2023. We explicitly filtered our research by excluding reviews, animal studies, and articles not published in the English language. We imported our research string using the Rayyan Intelligent Systematic Review Tool (https://www.rayyan.ai/, accessed on 3 January 2024) [8], and after duplicate removal, we obtained 424 articles available for double-blinded title and abstract screening by two independent reviewers (M.T., M.M.). Articles were excluded if they contained no neuroimaging data, if studies were not related to awareness, or if reviews or meta-analysis were presented. Once the reviewers reached a decision for all articles, they shared their decisions and discussed each conflicting case to reach a consensus. Finally, after additional relevant publications were identified from full-text bibliographies, 8 articles were considered eligible for the aim of this study and were included in the present review: 6 articles investigated unawareness in PD, and 2 did so in HD (Figure 1). To be sure that no studies had been incidentally excluded, we also performed a PubMed and Web of Science search with the terms “Parkinson’s” or “Huntington’s” to denote the disease of interest, combined with either “self-insight”, “anosognosia”, “awareness”, or “self-awareness” to denote the neuropsychological area of interest but without including “MRI”, “resonance”, “imaging”, or “PET” in order to identify all studies investigating awareness in PD and HD irrespective of neuroimaging data. From all studies identified (*n* = 2519), after the same screening and eligibility process already described for the primary search, we only identified studies including neuroimaging, and the same 8 previously identified neuroimaging studies emerged. This review was not pre-registered on PRISMA.

## 3. Results

Table 1 reports all studies included in this review. Participants’ characteristics, cognitive statuses (if available), domains of awareness investigated, awareness measurement used, neuroimaging techniques, and key findings were reported for each study.

### 3.1. Unawareness in Parkinson’s Disease

Six studies investigating brain correlates of impaired self-awareness in PD were included.

Maier et al. [15] were the first researchers to apply neuroimaging techniques to investigate unawareness in PD patients, focusing on unawareness of motor symptom measured by the discrepancy between a patient’s self-judgement after motor performance and clinicians’ judgements. By using FDG-PET, they found that not-demented PD patients with higher motor unawareness showed lower metabolism in the right inferior frontal gyrus during the OFF state. During the ON state, PD patients with higher motor unawareness had higher metabolism in the left inferior frontal gyrus, bilateral medial frontal gyrus, right superior frontal gyrus, and right precentral gyrus. They also found an association between unawareness of levodopa-induced dyskinesia and the metabolism of the midcingulate cortex and paracentral lobule, probably due to dopaminergic medication.

With a task eliciting the activation of the anterior cingulate (GO/NoGO task) in a task-based functional MRI study, Palermo et al. showed that impaired self-awareness of dyskinesia was associated with reduced functional recruitment of the cingulo-frontal (bilateral anterior cingulate cortex and right dorsolateral prefrontal cortex) and cingulo-opercular (bilateral anterior cingulate cortex and bilateral anterior insula cortex) regions in a cohort of patients chronically treated with dopaminergic medication [25]. Interestingly, the anterior cingulate cortex and anterior insula are considered major nodes of the “salience network” (SN) [31], and the dorsolateral prefrontal cortex is a principal region of the “frontoparietal network” [32,33].

A study investigating measures of social cognition and functional connectivity in different neurodegenerative diseases, including PD, showed that functional connectivity in the supramarginal gyrus, angular gyrus, and frontal pole correlated with performance in social cognition tasks but did not specifically investigate the association between brain connectivity regions and measures of unawareness in PD patients [26].

Considering only drug-naïve patients, Yoo et al. [27] showed that PD patients with unawareness of cognitive deficits had reduced white matter integrity, especially in the genu of the corpus callosum and anterior limb of the internal capsule, compared to those with preserved self-awareness.

In a recent study by Buchwitz et al. [28], from a larger cohort of 41 PD patients, a subgroup of 15 patients was evaluated via resting-state functional MRI to detect the association between measures of impaired self-awareness of motor symptoms (using the discrepancy score between self-reporting and a clinician’s judgment blinded to a patient’s answers, as described in [15]) and functional connectivity: they found that the ISAm total scores were positively associated with functional connectivity between the left inferior frontal gyrus and right insular cortex, right frontal operculum, and right frontal orbital cortex; significant negative correlations were found between ISAm for dyskinesia drug-induced and functional connectivity between the bilateral inferior frontal gyrus and right precentral gyrus, cingulate areas, supplementary motor area, and precuneus.

Finally, a study examining FDG-PET and cortical thickness correlates of ISAcog in PD patients found that ISAcog was associated with reduced metabolism in the bilateral superior medial frontal gyrus and midline structures, particularly in the anterior and midcingulate cortex [29].

### 3.2. Unawareness in Huntington’s Disease

Only two studies have investigated brain correlates of impaired self-awareness in HD.

Justo et al. [22] demonstrated in a cohort of early HD and preclinical carriers that anosognosia for motor impairment correlated with striatal atrophy. McCusker et al. [30] found the opposite pattern in a preclinical cohort of “unaware” people, classified by raters as showing very mild signs of HD. So-called “unaware” individuals had greater striatal and white matter volumes than “aware” subjects.

## 4. Discussion

Impaired self-awareness in PD and HD is still poorly understood, and there are no homogeneous results in terms of its brain correlates. Only six studies investigated neuroimaging features of unawareness in PD, and only two did so in HD; therefore, solid inference about neuropsychological or brain mechanisms cannot be achieved due to a lack of sufficient data. In addition, the great heterogeneity of studies features prevents adequate comparison between studies.

The clinical characteristics of the study participants, awareness domain investigated, measurement of awareness used, and in vivo neuroimaging technique applied are different across the studies and should be accurately considered prior to summarizing the results.

1. Participants’ clinical characteristics. Regarding PD studies, four studies included “not-demented” patients [15,25,27,29] and two studies also included PD patients with dementia [26,28], but the dementia inclusion criteria were not consistent: in one study, the CDR sum of boxes [34] was used [26], whereas in the other study, a PNDA assessment [35] was applied [28]. Different cognitive global severity assessments were considered when measuring cognitive decline: the Clinical Dementia Rating Scale sum of boxes [34]), Mini-Mental Scale Examination score [36], and MDS criteria [37] were used across studies. Also, the definition of Mild Cognitive Impairment varied across studies and was based on the MMSE score [27,28] or Litvan criteria [29,38]. Regarding HD studies, both studies [22,30] included data from not demented HD, early HD, or preclinical mutation carriers, but motor clinical symptoms differed in terms of severity and manifestation.
2. Awareness domain and methods of unawareness assessment. For PD studies, motor unawareness was investigated in three studies [15,25,28], whereas one study focused on social cognition [26] and two studies on general cognitive unawareness [27,29]. For motor symptoms, awareness for dyskinesia was evaluated in all three studies, whereas other motor symptoms, such as resting tremor or bradykinesia, were only considered in two studies [15,28]. In HD studies, only motor awareness was investigated, in particular chorea. Regarding methods of awareness assessment, various methods were used to quantify symptom awareness. Techniques used to evaluate awareness of motor functioning mainly included discrepancy judgment between self-reported and clinician ratings for both PD [15,25,28] and HD studies [22,30]; the study investigating social cognition [26] used the informant’s perspective of the subject’s self-concern, whereas studies of cognitive symptom awareness used self-reported interviews that were compared to objective cognitive performance [27] or healthy controls’ performances [29].
3. Neuroimaging techniques. Both functional and structural in vivo neuroimaging techniques were used. One study investigating motor awareness [15] and one study investigating cognitive awareness [29] in PD used FDG-PET. MRI, both with functional (seed-based resting state [26,28] or task-related [25]) and structural (DTI [26,27] or cortical thickness [27,29]) measures were used in studies investigating both motor and cognitive awareness in PD patients. Only structural MRI measures were investigated in HD studies.

Another important point to be considered when comparing studies in this review is the dopamine-related status at the time of the awareness evaluation. It is well known that dopaminergic therapies may impact cognitive and neuropsychiatric symptoms [39], as well neuroimaging functional activity [40]; therefore, the ON or OFF state during evaluation should be considered: the dopaminergic state varied across the studies, potentially interfering with the results. Thus, we decided to report and compare results across studies separately for the OFF or drug-naïve state and the ON state.

### 4.1. Neural Correlates in PD in OFF State or Drug-Naïve Patients

**Figure 2:**
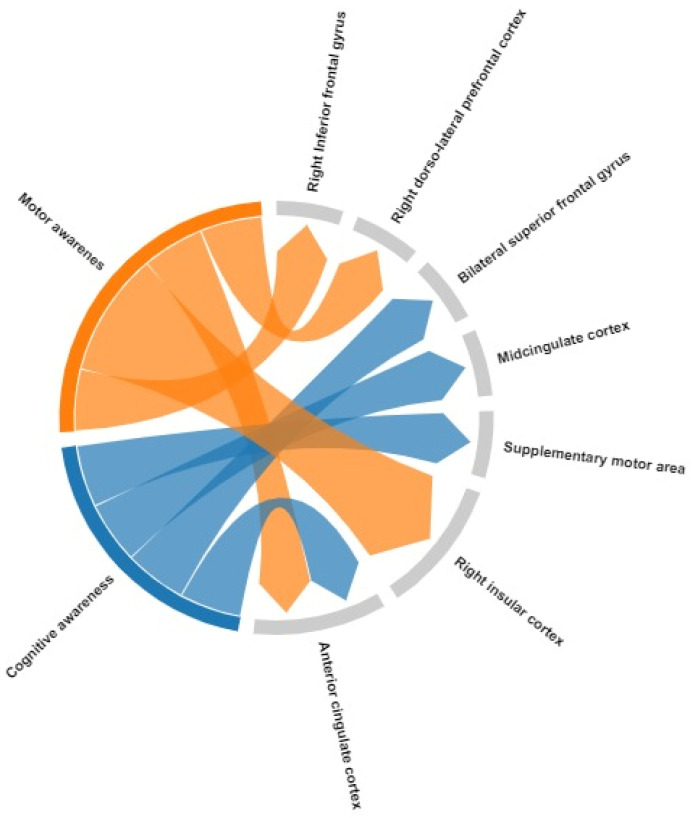
Graphical representation of cortical regions involved in motor and cognitive awareness in PD patients (OFF state or drug-naïve patients). Chord size depicts the number of studies in which the specific brain region was found to be associated with unawareness.

When considering only PD studies evaluating neural correlates of unawareness in the absence of significant dopaminergic effect (drug-naïve [27] or OFF state [15,25,29]), more consistent result emerged. As a matter of fact, impaired self-awareness for motor or cognitive symptoms was mainly found to be associated with structural and/or functional alterations in the frontal, anterior insular, and anterior–midcingulate cortex. More precisely, (i) reduced FDG-PET metabolism in the inferior frontal gyrus [15], superior medial frontal gyrus, and anterior cingulate cortex [29]; (ii) reduced MRI functional activation in the anterior cingulate cortex, anterior insular, and dorsolateral prefrontal cortex [25]; and (iii) reduced white matter integrity in genu of corpus callosum and anterior limb of internal capsule [27] were found to be associated with self-impaired awareness, even if heterogeneous methods of awareness assessment and neuroimaging techniques were used. All these regions are known to be involved in executive-monitoring processing; in particular, the dorsolateral prefrontal cortex is a principal region of the “executive-control network” [32], while anterior cingulate and anterior insula are major nodes of the “salience network” [31]. These networks are involved in controlling or coordinating multiple domains of cognitive and executive functioning, which enable the abilities of self-monitoring and feedback integration and are known to be involved in unawareness and anosognosia in AD patients [41]. The inferior frontal gyrus is known to be involved in interoception, social cognition, and emotion [42], which serves as a sensory–cognitive integration area for the frontoparietal network [32]. When considering motor and cognitive self-impaired awareness separately (Figure 2, Table 2), the results seem to confirm the involvement of attentional and saliences regions and the interplay between them. Only two studies explored cognitive awareness domain in drug-naïve [27] or OFF state [29] patients: Maier et al. found significant cortical region involvement (bilateral superior medial frontal gyrus, anterior cingulate cortex, midcingulate cortex, and supplementary motor area) in cognitive unawareness, whereas the disruption of matter integrity of midline regions in the anterior corpus callosum and anterior limb of internal capsule was demonstrated in the study of Yoo et al. [27]. There results seem to converge, highlighting the major contribution of the regions interconnected within salience network to cognitive awareness, in line with findings for other neurodegenerative diseases, such as AD [41]. Regarding the motor awareness domain, alterations in functional or structural measures in inferior frontal gyrus, insular cortex, anterior cingulate cortex, and dorsolateral prefrontal cortex were found [15,25], confirming the potential interplay between salience and frontoparietal networks.

### 4.2. Neural Correlates in PD in ON State

**Figure 3:**
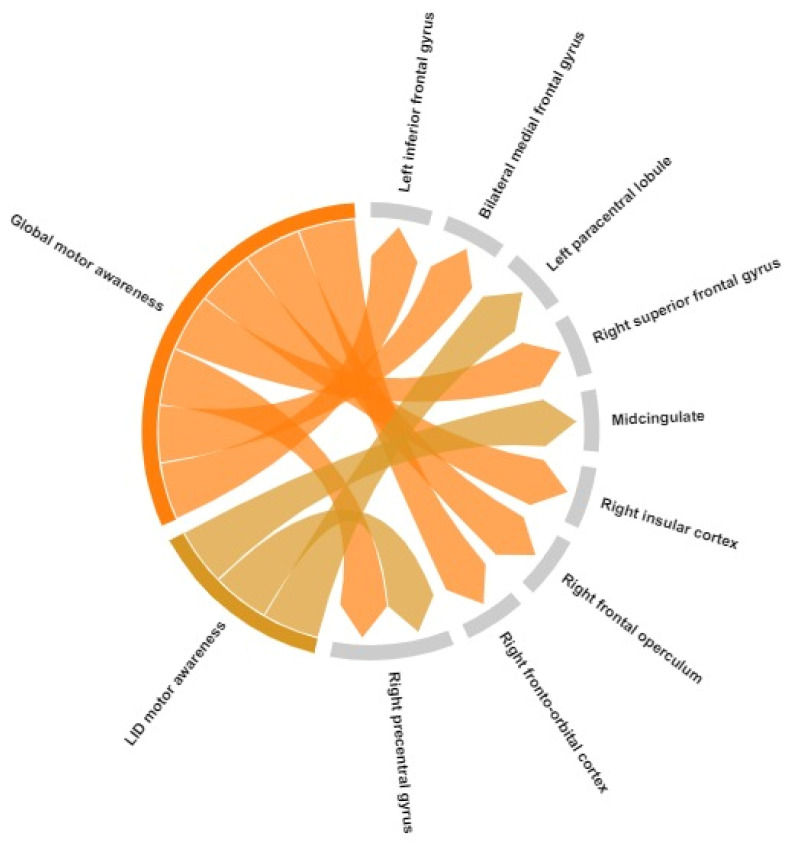
Graphical representation of cortical regions involved in motor awareness in PD patients (ON state). LID = levodopa-induced dyskinesia. Chord size depicts the number of studies in which the specific brain region was found to be associated with unawareness.

When considering PD studies evaluating neural correlates of awareness in ON drug state, thus influenced by dopaminergic activation (Table 3 and Figure 3), studies reported a positive association between motor unawareness and (i) increased metabolic activation in the left inferior frontal gyrus, bilateral medial frontal gyrus, right superior frontal gyrus, and right precentral gyrus for the total score of motor awareness [15], (ii) positive correlation between dyskinesia induced by dopamine and metabolism in bilateral midcingulate cortex and paracentral lobule [15], and (iii) increased functional connectivity between the left inferior frontal gyrus and right insular cortex, frontal operculum, and right fronto-orbital cortex for global motor symptoms [28]. A negative association was demonstrated between ISAm-LID scores and functional connectivity between both the inferior frontal gyrus and right precentral gyrus [28]. The results of Multani et al. were not considered in this section because no specific PD neuroimaging correlates of awareness measures (RSMS) were described. Divergent results are probably related to effects of dopamine medication, and differences must be interpreted with caution. Impaired self-awareness of LID has previously been related to deficits in executive functioning due to the dopaminergic overstimulation of mesolimbic circuits [16,17]. Indeed, according to a physiopathological hypothesis, Vitale and coauthors suggested that dopaminergic overstimulation in PD, while inducing dyskinesias by acting on basal ganglia, may affect the awareness of involuntary movements by stimulating mesocorticolimbic pathways [43], and this mechanism may be reflected in the overactivation of these areas detectable using in vivo neuroimaging techniques. No studies reported analyses about association between cognitive awareness and brain correlated in ON dopamine state. 

### 4.3. Neural Correlates in HD

In HD, associations between awareness of motor impairment and brain measures emerged as primarily dependent on the severity of the motor disorders. Nevertheless, when controlling for severity of the motor disorder, striatal atrophy (left and right caudate and putamen) remained significant [22]. Even if apparently opposite, McCusker et al. [30] found that the so-called “unaware” individuals had greater striatal and white matter volumes than an “aware” subgroup. However, the “unaware” group also had better motor function (speeded tapping) and fewer functional limitations, suggesting that they were less overtly affected than the aware group; therefore, striatal atrophy in the aware group could be an expression of more advanced disease stage, and inferences about awareness association should be taken cautiously.

### 4.4. Limitations

Some limitations should be noted. We focused the search only on peer-reviewed studies within language limits, possibly increasing the risk of publication bias. Furthermore, considering methodological issues, we are aware that there is considerable heterogeneity in the awareness assessment tools used in each study, and it is well known that different measures may lead us to explore different aspects of awareness [7]. As a matter of fact, awareness is not an all-or-nothing phenomenon; it may be that patients are aware of some type of impairment but not of others. Whereas measurements based on an examiner’s ratings usually refer to global and general unawareness (including cognitive, behavioral, and functional abilities), measurements of discrepancy and performance variably refer to different specific domains depending on the questionnaire and neuropsychological tests adopted. All these approaches have significant limitations, and there is no consensus on the most suitable method to determine anosognosia in dementia. Therefore, different measures of awareness may translate into different neural correlates and brain region activations. In addition, not all studies accurately reported the cognitive statuses of their participants, and only some studies applied common clinical criteria for mild cognitive impairment or dementia status. Thus, interpreting study findings becomes very challenging when a mixed population of cognitively normal people, mildly impaired people, and those with dementia are included.

## 5. Conclusions

The findings from the literature investigating brain correlates of awareness of cognitive and motor symptoms in people with PD and HD are mixed, limited, and influenced by methodological heterogeneity. Nevertheless, a pattern involving regions of salience and frontoparietal networks seems to emerge from studies of PD patients. Still, little data are available for HD patients to infer any conclusion. Studies of awareness have mainly focused on Alzheimer’s Disease (AD) patients, and models of anosognosia have mainly been proposed to interpret awareness in memory and metacognitive domains [9,13,44,45,46], suggesting that self-awareness and anosognosia rely upon different networks that are functionally correlated and support communication between an “external” source of information and “internal” personal knowledge. In the AD literature, emerging evidence suggests that awareness may rely upon an interplay between the salience network, a resting-state network known to be involved in detecting, processing, and integrating internally and externally generated salient information [31]; the default mode network, a set of interconnected brain regions that are suppressed during tasks that demand externalized attention [47]; and the fronto-parietal network, a network known to be implicated in a wide range of executive functions, including working memory, performance monitoring, and planning [48,49]. Considering that, in our review, anterior cingulate cortex, midcingulate cortex, dorsolateral prefrontal cortex, and insula emerged as key regions implicated in awareness in PD, it is plausible that the same dysfunctional network dynamics seen in AD patients may also be involved in anosognosia in PD patients. These network changes should be, therefore, considered markers of a symptom rather than markers of a specific disease. Future studies of resting-state functional magnetic resonance imaging are needed to test this hypothesis by investigating large-scale neurocognitive networks in PD and HD patients. Future studies based on the integration of multimodal neuroimaging techniques with MRI-based estimates of dopaminergic neurotransmitter systems are also needed to better understand the contribution of dopaminergic balance to awareness. Finally, given the chronic nature of PD and HD and the high impact of impaired self-awareness on patients and caregivers, longitudinal studies are also needed to accurately establish the neural underpinnings of symptom awareness along with disease progression.

## Figures and Tables

**Table 1:** Studies of impaired self-awareness brain correlates in PD and HD.

Study	Participant	Clinical and Cognitive Status	Domain of Awareness	Awareness Assessment	Neuroimaging Technique	Key Neuroimaging Findings
Maier et al., 2016 [15]	31 PD - Median age of 66 years - Median disease duration of 9 years	- Not demented (MMSE > 26) - Not depressed (BDI-II < 19)	Motor awareness (dyskinesia, resting tremor, bradykinesia)	ISAm-PD (discrepancy score between self-reporting and clinician’s judgment blinded to patient’s answers)	FDG-PET: - correlation analyses between metabolism and measures of awareness in OFF and ON drug condition	OFF state: negative correlation between FDG-PET metabolism and ISAm total score in right inferior frontal gyrus (*p* = 0.016) and right insular cortex (*p* = 0.083). ON state: positive correlation between ISAm total score and metabolism in left inferior frontal gyrus, bilateral medial frontal gyrus, right superior frontal gyrus, and right precentral gyrus (*p* < 0.05); positive correlation between ISAm-LID and metabolism in bilateral midcingulate cortex and left paracentral lobule (*p* < 0.05)
Palermo et al., 2018 [25]	27 PD - Mean age of 64 years - Median disease duration of 10 years	- Not demented (MMSE > 26) - Good clinical response to medication - Presence of motor fluctuations	Motor awareness (dyskinesia)	Dyskinesia Index as a discrepancy score between patient’s judgement and that of clinician	Task-related fMRIGo-NoGO paradigm: - correlation analyses between neuroimaging measures and measures of awareness - therapeutic washout during neuroimaging acquisition (last medication 8 h before MRI)	Dyskinesia unawareness negatively correlated with the NoGO/GO response in bilateral anterior cingulate cortex, bilateral anterior insular cortex, and right dorsolateral prefrontal cortex (*p* < 0.05)
Multani et al., 2019 [26]	57 participants (FTD = 10, AD = 18, PD = 19, and HC = 10) - Mean PD age of 70 years - Median disease duration not reported	Mean CDR-SoB in PD group = 2.9	Social Cognition	Revised Self- Monitoring Scale (RSMS) as a measure of the subjects’ awareness of their own social behavior (as assessed by the informant)	Resting-state fMRI (seed-based) + Diffusion Tensor Imaging and Structural Connectivity: - group-comparison analysis - ON drug state	Positive correlation between RSMS and right supramarginal gyrus, posterior division, right angular gyrus, and right frontal pole (*p* = 0.003); functional connectivity across all groups, but data of PD separately were not mentioned
Yoo et al., 2021 [27]	153 drug-naïveand non-dementedPD patients - Mean age of 64 years - Median disease duration of 1.6 years	Cognitive intact or MCI	Cognitive symptoms	Cognitive subjective complaint interview compared to objective performance (impaired self-awareness of cognitive deficits, IACd): patients were grouped in the PD-IACd+ (with IACd) and PD-IACd- (without IACd) groups	MRI cortical thickness, diffusion tensor imaging (DTI) and striatal dopamine transporter (DAT): - group-comparison analysis - drug-naïve	Patients in the PD-IACd+ group had white matter disintegrity, especially in genu of the corpus callosum and anterior limb of internal capsule, compared to those without IACd; cortical thickness or striatal DAT availability was comparable regardless of presence of IACd. Amongst patients with mild cognitive impairment, those with IACd had more severe WM disintegrity than those without IACd
Buchwitz et al., 2023 [28]	15 PD - Mean age of 64 years - Disease duration not reported	- Not severely demented (PNDA > 15) - Not severely depressed (BDI-II < 28)	Motor awareness (dyskinesia, resting tremor, bradykinesia)	ISAm-PD (discrepancy score between self-reporting and clinician’s judgment blinded to patient’s answers)	Resting-state fMRI (seed-based): - ON drug state	ISAm total score were positively associated with functional connectivity between left inferior frontal gyrus and right insular cortex, right frontal operculum, and right frontal orbital cortex; ISAm-LID scores were negatively correlated with functional connectivity between both inferior frontal gyrus and right precentral gyrus
Maier et al., 2023 [29]	63 PD and 30 HC - Mean age of 68 years - Median disease duration of 5 years	Cognitive intact or PD-MCI - Not demented (MDS criteria) - Not depressed (BDI-II < 21)	Cognitive awareness	ISAcog constructed using the Cognitive Failures Questionnaire (z scores comparison with HC)	FDG-PET + MRI cortical thickness on brain regions where metabolism correlated negatively with ISAcog: - correlation analyses between brain regions and ISAcog - OFF drug state	FDG-PET findings: negative correlation between ISAcog and metabolism in bilateral superior medial frontal gyrus, anterior and midcingulate cortex (*p* < 0.001); MRI findings: no correlation with cortical thickness in regions found with PET
Justo et al., 2013 [22]	28 early HD and 28 preclinical HD carriers and 12 HC - Mean Age HD of 51 years Carrier of 40 years - disease duration not reported	Early HD not demented but with chorea	Motor awareness (chorea)	Ad hoc questionnaire investigating (A) insight into motor dysfunction, (B) identification of the cause, (C) perception of the practical consequences of the motor dysfunction, and (D) concurrent awareness of involuntary movements	Structural MRI: - correlation analyses between brain structures and insight-related scores	Positive correlation between insight and striatal volumes (when controlled for the severity of motor disorders)
McCusker et al., 2013 [30]	550 HD mutation carriers Age range of 42–46 years	Not demented	Motor awareness	Unawareness was identified when no motor symptoms were self-reported but when a diagnosis of definite motor HD was made.	Structural MRI: - group comparison analyses between aware and unaware patients	Unaware group had greater striatal and white matter volumes compared to aware group

PD: Parkinson’s Disease; HD: Huntington’s Disease; HC: healthy controls; MMSE: Mini-Mental State Examination; BDI: Beck Depression Inventory; MRI: magnetic resonance imaging; FDG-PET: 18-F fluorodeoxyglucose positron emission tomography; ys: years; ISAcog: impaired self-awareness in cognitive domain; ISAm: impaired self-awareness in motor domain; CDR-SoB: clinical dementia rating scale, sum of boxes; MDS: Movement Disorder Society Clinical Diagnostic Criteria for Parkinson’s Disease; PNDA: Parkinson’s Neuropsychometric Dementia Assessment; IACd: impaired self-awareness of cognitive deficits.

**Table 2:** Cortical brain regions involved in motor and cognitive awareness in PD patients (OFF state or drug-naïve patients).

Domain	Brain Region	Direction of Association with Awareness Measure	Study
Motor awareness	Right Inferior frontal gyrus	Negative association	Maier et al., 2016 [15]
	Right insular cortex	Negative association	Maier et al., 2016 [15]; Palermo et al., 2018 [25]
	Bilateral anterior cingulate cortex	Negative association	Palermo et al., 2018 [25]
	Right dorso-lateral prefrontal cortex	Negative association	Palermo et al., 2018 [25]
Cognitive awareness	Bilateral superior frontal gyrus	Negative association	Maier et al., 2023 [29]
	Anterior cingulate cortex	Negative association	Maier et al., 2023 [29]
	Midcingulate cortex	Negative association	Maier et al., 2023 [29]
	Supplementary motor area	Negative association	Maier et al., 2023 [29]

**Table 3:** Cortical brain regions involved in motor awareness in PD patients (ON state). For [28], regions reported are those emerged from MRI connectivity analyses with inferior frontal gyrus as functional connectivity seed.

Domain	Brain Region	Direction of Association with Awareness Measure	Study
Motor awareness	Left inferior frontal gyrus	Positive association	Maier et al., 2016 [15]
	Bilateral medial frontal gyrus	Positive association	Maier et al., 2016 [15]
	Left paracentral lobule	Positive association	Maier et al., 2016 [15]
	Right precentral gyrus	Positive association	Maier et al., 2016 [15]
	Right superior frontal gyrus	Positive association	Maier et al., 2016 [15]
	Midcingulate	Positive association	Maier et al., 2016 [15]
	Right insular cortex	Positive association	Buchwitz et al., 2023 [28]
	Right frontal operculum	Positive association	Buchwitz et al., 2023 [28]
	Right fronto-orbital cortex	Positive association	Buchwitz et al., 2023 [28]
	Right precentral gyrus	Negative association	Buchwitz et al., 2023 [28]

## References

[1] Rice D.G., Greenfield N.S. (1969). Psychophysiological correlates of la belle indifference. Arch. Gen. Psychiatry.

[2] Babinski J. (1914). Contribution a l’étude des troubles mentaux dans hémiplégie organique cèrébrale (anosognosie). Rev. Neurol..

[3] McGlynn S.M., Schacter D.L. (1989). Unawareness of deficits in neuropsychological syndromes. J. Clin. Exp. Neuropsychol..

[4] Prigatano G.P. (2009). The Study of Anosognosia.

[5] Aalten P., van Valen E., Clare L., Kenny G., Verhey F. (2005). Awareness in dementia: A review of clinical correlates. Aging Ment. Health.

[6] Aalten P., van Valen E., de Vugt M.E., Lousberg R., Jolles J., Verhey F.R. (2006). Awareness and behavioral problems in dementia patients: A prospective study. Int. Psychogeriatr..

[7] Tondelli M., Barbarulo A.M., Vinceti G., Vincenzi C., Chiari A., Nichelli P.F., Zamboni G. (2018). Neural Correlates of Anosognosia in Alzheimer’s Disease and Mild Cognitive Impairment: A Multi-Method Assessment. Front. Behav. Neurosci..

[8] Zamboni G., Wilcock G. (2011). Lack of awareness of symptoms in people with dementia: The structural and functional basis. Int. J. Geriatr. Psychiatry.

[9] Kaszniak A.W., Edmons E.C., Prigatano G.P. (2010). Anosognosia and Alzheimer’s Disease: Behavioral studies. The Study of Anosognosia.

[10] Lenzoni S., Morris R.G., Mograbi D.C. (2020). The Petrified Self 10 Years After: Current Evidence for Mnemonic anosognosia. Front. Psychol..

[11] Leocadi M., Canu E., Paldino A., Agosta F., Filippi M. (2023). Awareness impairment in Alzheimer’s disease and frontotemporal dementia: A systematic MRI review. J. Neurol..

[12] Mondragon J.D., Maurits N.M., De Deyn P.P., Alzheimer’s Disease Neuroimaging I. (2021). Functional connectivity differences in Alzheimer’s disease and amnestic mild cognitive impairment associated with AT(N) classification and anosognosia. Neurobiol. Aging.

[13] Zamboni G., Drazich E., McCulloch E., Filippini N., Mackay C.E., Jenkinson M., Tracey I., Wilcock G.K. (2013). Neuroanatomy of impaired self-awareness in Alzheimer’s disease and mild cognitive impairment. Cortex.

[14] Maier F., Prigatano G.P., Kalbe E., Barbe M.T., Eggers C., Lewis C.J., Burns R.S., Morrone-Strupinsky J., Moguel-Cobos G., Fink G.R. (2012). Impaired self-awareness of motor deficits in Parkinson’s disease: Association with motor asymmetry and motor phenotypes. Mov. Disord..

[15] Maier F., Williamson K.L., Tahmasian M., Rochhausen L., Ellereit A.L., Prigatano G.P., Kracht L., Tang C.C., Herz D.M., Fink G.R. (2016). Behavioural and neuroimaging correlates of impaired self-awareness of hypo- and hyperkinesia in Parkinson’s disease. Cortex.

[16] Amanzio M., Monteverdi S., Giordano A., Soliveri P., Filippi P., Geminiani G. (2010). Impaired awareness of movement disorders in Parkinson’s disease. Brain Cogn..

[17] Amanzio M., Palermo S., Zibetti M., Leotta D., Rosato R., Geminiani G., Lopiano L. (2014). Self-unawareness of levodopa induced dyskinesias in patients with Parkinson’s disease. Brain Cogn..

[18] Pennington C., Duncan G., Ritchie C. (2020). Altered awareness of motor symptoms in Parkinson’s disease and Dementia with Lewy Bodies: A systematic review. Int. J. Geriatr. Psychiatry.

[19] Pennington C., Duncan G., Ritchie C. (2021). Altered awareness of cognitive and neuropsychiatric symptoms in Parkinson’s disease and Dementia with Lewy Bodies: A systematic review. Int. J. Geriatr. Psychiatry.

[20] Sitek E.J., Thompson J.C., Craufurd D., Snowden J.S. (2014). Unawareness of deficits in Huntington’s disease. J. Huntingt. Dis..

[21] Hergert D.C., Sanchez-Ramos J., Cimino C.R. (2020). Awareness of Chorea in Huntington’s Disease. J. Huntingt. Dis..

[22] Justo D., Charles P., Daunizeau J., Delmaire C., Gargiulo M., Hahn-Barma V., Naccache L., Durr A. (2013). Is non-recognition of choreic movements in Huntington disease always pathological?. Neuropsychologia.

[23] Maier F., Prigatano G.P. (2017). Impaired Self-Awareness of Motor Disturbances in Parkinson’s Disease. Arch. Clin. Neuropsychol..

[24] McCusker E., Loy C.T. (2014). The many facets of unawareness in huntington disease. Tremor Other Hyperkinet. Mov..

[25] Palermo S., Lopiano L., Morese R., Zibetti M., Romagnolo A., Stanziano M., Rizzone M.G., Geminiani G.C., Valentini M.C., Amanzio M. (2018). Role of the Cingulate Cortex in Dyskinesias-Reduced-Self-Awareness: An fMRI Study on Parkinson’s Disease Patients. Front. Psychol..

[26] Multani N., Taghdiri F., Anor C.J., Varriano B., Misquitta K., Tang-Wai D.F., Keren R., Fox S., Lang A.E., Vijverman A.C. (2019). Association between Social Cognition Changes and Resting State Functional Connectivity in Frontotemporal Dementia, Alzheimer’s Disease, Parkinson’s Disease, and Healthy Controls. Front. Neurosci..

[27] Yoo H.S., Kwon H., Chung S.J., Sohn Y.H., Lee J.M., Lee P.H. (2021). Neural correlates of self-awareness of cognitive deficits in non-demented patients with Parkinson’s disease. Eur. J. Neurol..

[28] Buchwitz T.M., Ruppert-Junck M.C., Greuel A., Maier F., Thieken F., Jakobs V., Eggers C. (2023). Exploring impaired self-awareness of motor symptoms in Parkinson’s disease: Resting-state fMRI correlates and the connection to mindfulness. PLoS ONE.

[29] Maier F., Greuel A., Hoock M., Kaur R., Tahmasian M., Schwartz F., Csoti I., Jessen F., Drzezga A., van Eimeren T. (2023). Impaired self-awareness of cognitive deficits in Parkinson’s disease relates to cingulate cortex dysfunction. Psychol. Med..

[30] McCusker E.A., Gunn D.G., Epping E.A., Loy C.T., Radford K., Griffith J., Mills J.A., Long J.D., Paulsen J.S., Group P.-H.I.o.t.H.S. (2013). Unawareness of motor phenoconversion in Huntington disease. Neurology.

[31] Seeley W.W., Menon V., Schatzberg A.F., Keller J., Glover G.H., Kenna H., Reiss A.L., Greicius M.D. (2007). Dissociable intrinsic connectivity networks for salience processing and executive control. J. Neurosci..

[32] Uddin L.Q., Yeo B.T.T., Spreng R.N. (2019). Towards a Universal Taxonomy of Macro-scale Functional Human Brain Networks. Brain Topogr..

[33] Dosenbach N.U., Fair D.A., Miezin F.M., Cohen A.L., Wenger K.K., Dosenbach R.A., Fox M.D., Snyder A.Z., Vincent J.L., Raichle M.E. (2007). Distinct brain networks for adaptive and stable task control in humans. Proc. Natl. Acad. Sci. USA.

[34] Morris J.C. (1993). The Clinical Dementia Rating (CDR): Current version and scoring rules. Neurology.

[35] Kalbe E., Calabrese P., Kohn N., Hilker R., Riedel O., Wittchen H.U., Dodel R., Otto J., Ebersbach G., Kessler J. (2008). Screening for cognitive deficits in Parkinson’s disease with the Parkinson neuropsychometric dementia assessment (PANDA) instrument. Park. Relat. Disord..

[36] Folstein M.F., Folstein S.E., McHugh P.R. (1975). “Mini-mental state”. A practical method for grading the cognitive state of patients for the clinician. J. Psychiatr. Res..

[37] Emre M., Aarsland D., Brown R., Burn D.J., Duyckaerts C., Mizuno Y., Broe G.A., Cummings J., Dickson D.W., Gauthier S. (2007). Clinical diagnostic criteria for dementia associated with Parkinson’s disease. Mov. Disord..

[38] Litvan I., Goldman J.G., Troster A.I., Schmand B.A., Weintraub D., Petersen R.C., Mollenhauer B., Adler C.H., Marder K., Williams-Gray C.H. (2012). Diagnostic criteria for mild cognitive impairment in Parkinson’s disease: Movement Disorder Society Task Force guidelines. Mov. Disord..

[39] Mattay V.S., Tessitore A., Callicott J.H., Bertolino A., Goldberg T.E., Chase T.N., Hyde T.M., Weinberger D.R. (2002). Dopaminergic modulation of cortical function in patients with Parkinson’s disease. Ann. Neurol..

[40] Clos M., Bunzeck N., Sommer T. (2019). Dopamine is a double-edged sword: Dopaminergic modulation enhances memory retrieval performance but impairs metacognition. Neuropsychopharmacology.

[41] Valera-Bermejo J.M., De Marco M., Venneri A. (2021). Altered Interplay Among Large-Scale Brain Functional Networks Modulates Multi-Domain Anosognosia in Early Alzheimer’s Disease. Front. Aging Neurosci..

[42] Adolfi F., Couto B., Richter F., Decety J., Lopez J., Sigman M., Manes F., Ibanez A. (2017). Convergence of interoception, emotion, and social cognition: A twofold fMRI meta-analysis and lesion approach. Cortex.

[43] Vitale C., Pellecchia M.T., Grossi D., Fragassi N., Cuomo T., Di Maio L., Barone P. (2001). Unawareness of dyskinesias in Parkinson’s and Huntington’s diseases. Neurol. Sci..

[44] Agnew A.N., Morris R.G. (1998). The heteogeneity of anosognosia for memory impairment in Alzheimer’s disease: A review of the literature and a proposed model. Aging Ment. Health.

[45] Morris R.G., Hannesdottir K., Oxford University Press (2004). Loss of “awareness” in Alzheimer’s disease. Cognitive Neuropsychology of Alzheimer’s Disease.

[46] Salmon E., Meyer F., Genon S., Collette F., Bastin C. (2023). Neural correlates of impaired cognitive processes underlying self-unawareness in Alzheimer’s disease. Cortex.

[47] Raichle M.E., MacLeod A.M., Snyder A.Z., Powers W.J., Gusnard D.A., Shulman G.L. (2001). A default mode of brain function. Proc. Natl. Acad. Sci. USA.

[48] Niendam T.A., Laird A.R., Ray K.L., Dean Y.M., Glahn D.C., Carter C.S. (2012). Meta-analytic evidence for a superordinate cognitive control network subserving diverse executive functions. Cogn. Affect. Behav. Neurosci..

[49] Spreng R.N., Stevens W.D., Chamberlain J.P., Gilmore A.W., Schacter D.L. (2010). Default network activity, coupled with the frontoparietal control network, supports goal-directed cognition. Neuroimage.

